# [18F]FDG PET/CT versus [18F]FDG PET/MRI for the diagnosis of colorectal liver metastasis: A systematic review and meta-analysis

**DOI:** 10.3389/fonc.2023.1114059

**Published:** 2023-02-13

**Authors:** Zhi Miao, Xiaomeng Zhao, Xuanwen Li

**Affiliations:** ^1^ Frontier Science Center for Synthetic Biology and Key Laboratory of Systems Bioengineering (Ministry of Education), Tianjin University, Tianjin, China; ^2^ School of Chemical Engineering and Technology, Collaborative Innovation Center of Chemical Science and Engineering (Tianjin), Tianjin University, Tianjin, China; ^3^ Graduate School of Health Science, Suzuka University of Medical Science, Suzuka, Japan

**Keywords:** PET/CT, PET/MRI, colorectal neoplasms, liver metastasis, meta-analysis

## Abstract

**Purpose:**

The purpose of our meta-analysis and systematic review was to compare the diagnostic performance of [18F]FDG PET/CT and [18F]FDG PET/MRI in colorectal liver metastasis.

**Methods:**

We searched PubMed, Embase, and Web of Science for eligible articles until November 2022. Studies focusing on the diagnostic value of [18F]FDG PET/CT or PET/MRI for colorectal liver metastasis were included. Using a bivariate random-effect model, the pooled sensitivity and specificity for [18F]FDG PET/CT and [18F]FDG PET/MRI were reported as estimates with 95% confidence intervals (CIs). Heterogeneity among pooled studies was assessed using the I^2^ statistic. The Quality Assessment of Diagnostic Performance Studies (QUADAS-2) method was used to evaluate the quality of the studies that were included.

**Results:**

There were a total of 2743 publications identified in the initial search, finally, a total of 21 studies comprising 1036 patients were included. The pooled sensitivity, specificity, and AUC of [18F]FDG PET/CT in were 0.86 (95% CI: 0.76-0.92), 0.89 (95% CI: 0.83-0.94), and 0.92(95% CI: 0.90-0.94). [18F]FDG PET/MRI were 0.84 (95% CI: 0.77-0.89), 1.00 (95% CI: 0.32–1.00), and 0.89(95% CI: 0.86-0.92), respectively.

**Conclusion:**

[18F]FDG PET/CT shows similar performance compared to [18F]FDG PET/MRI in detecting colorectal liver metastasis. However, pathological results were not obtained for all patients in the included studies and PET/MRI results were derived from studies with small sample sizes. There is a need for additional, larger prospective studies on this issue.

**Systematic review registration:**

https://www.crd.york.ac.uk/prospero/, identifier (CRD42023390949).

## 1 Introduction

With a 50-60% prevalence, the liver is the most significant metastatic location from colorectal cancer (CRC) (1, 2). Metastases are restricted to the liver in about one-third of these individuals at the time of detection, increasing curative therapy options (3, 4). Complete resection is a common treatment option for liver metastases, which is beneficial to the prognosis (5). This observation highlights the importance of correct staging or restaging of CRC for a personalized therapy decision.

Several diagnostic imaging modalities, including contrast-enhanced CT, MRI, and contrast-enhanced ultrasonography (US), are currently available for CRC staging or restaging. Contrast-enhanced CT is still considered the standard imaging modality for CRC staging and restaging, however, recent studies have revealed that MRI offers superior sensitivity for detecting liver metastases (6) contrast-enhanced US is a technique that depends a lot on the operator, which may explain the lower reported liver metastasis detection sensitivity (7). There have been reports of improved sensitivity with intraoperative or laparoscopic ultrasound, but this method cannot be performed without invasive procedures (8). As a result, the most effective imaging approach has not yet been defined.

Positron emission tomography/computed tomography (PET/CT) is an established modality for evaluating local recurrence or distant metastasis of colorectal cancer, which provides specific molecular and metabolic information (9–11). In terms of tumor staging, hybrid PET/CT system improves lesion localization and interpretation of colorectal cancer compared to PET or CT alone (12). Numerous studies have shown that PET/CT has unique advantages over conventional methods for colorectal liver metastasis (13–16). However, during the past decade, radionuclide imaging techniques such as hybrid positron emission tomography/magnetic resonance imaging (PET/MRI) have attracted attention as they allow well detection of cancer metastasis. Head-to-head comparison by Brendle et al. showed that PET/CT had lower sensitivity but better specificity than PET/MRI, however, the diagnostic performance of PET/CT was lower than previous studies due to the high percentage of mucinous tumors and limited spatial resolution (17). Nowadays, although many studies have reported the good performance of hybrid [18F]FDG PET/CT in colorectal liver metastasis, few have quantitatively assessed its relative performance compared to hybrid [18F]FDG PET/MRI.

Therefore, in the current study, we aimed to perform a meta-analysis by searching all available literature to obtain the diagnostic performance of hybrid [18F]FDG PET/CT and hybrid [18F]FDG PET/MRI modality in the diagnosis of colorectal liver metastasis.

## 2 Manuscript formatting

### 2.1 Material and methods

Our study protocol was registered on PROSPERO (CRD42023390949). This study was conducted according to the Preferred Reporting Items for Systematic Reviews and Meta-Analyses guidelines (**Supplementary Table 1**) (18).

#### 2.1.1 Search strategy

A comprehensive search was conducted of the PubMed, Embase, and Web of Science databases for all available literatures through November, 2022 based on the following combination of terms (1): colon OR colorectal OR rectal (2); PET-MRI OR PET-MR OR Positron Emission Tomography Magnetic Resonance Imaging OR Positron Emission Tomography Computed Tomography OR PET/CT (3); liver metastasis OR liver metastases. Studies that might have been relevant were also included from the reference lists.

#### 2.1.2 Inclusion and exclusion criteria

Only studies that met all of the following criteria were included (1): articles evaluating the diagnostic performance of [18F]FDG PET/CT or [18F]FDG PET/MRI for colorectal liver metastasis (2); number of patients or lesions ≥ 10 (3); histological pathology or follow-up imaging as gold standard. The exclusion criteria were (1): Irrelevant topic (2); duplicated articles (3); case reports, abstract, letters, review, or meta-analysis (4); true positive (TP), false positive (FP), true negative (TN), false negative (FN) data could not be extracted. After evaluating the titles and abstracts of the articles according to the inclusion and exclusion criteria, the full-text versions of the selected articles were examined to determine if they met the inclusion criteria. Disagreements among the researchers were settled through consensus.

#### 2.1.3 Quality assessment and data extraction

Using the Quality Assessment of Diagnostic Performance Studies (QUADAS-2) technique, two researchers independently assessed the quality of the included studies. Each study’s risk of bias and applicability were evaluated. It includes four important domains, including (1) patient selection (2); index test (3); reference standard; and (4) the flow and timing (19). For risk of bias, the question for patient selection was if consecutive patients enrolled; The question for the index test was if the index test results were interpreted without knowledge of the results of the reference standard; The question for reference standard was if the reference standard results interpreted without knowledge of the results of the index test; The question for the flow and timing was if there an appropriate interval between index tests and the reference(3 months). For application concern, the question for patient selection was if concerns that the included patients do match the review question; The question for index test was if there were concerns that the index test, its conduct, or its interpretation differ from the review question; The question for reference standard was if there concerns that the target condition as defined by the reference standard does not match the review question. The evaluation of each study was rated as high, low, or unclear in terms of risk of bias and applicability. To settle any potential disagreements, a third reviewer was engaged in. RevMan (version 5.3) was used for the analysis.

Data extraction for all included papers was conducted separately by two researchers. The data that were extracted included (1): the author, year of publication (2); study characteristics including country, study design, analysis, reference standard (3); patient characteristics including number of patients, clinical indication, mean/median age, chemotherapy before PET (4); technical characteristics including types of imaging tests, scanner modality, ligand dose, time from injection to acquisition, image analysis, TP, FP, FN, TN. Data were manually retrieved from the literature, tables, and figures when not clearly stated. When the paper lacked sufficient information, we contacted the corresponding authors *via* email to request further data or clarification. Two researchers addressed their disagreements through consensus.

#### 2.1.4 Data synthesis and statistical analysis

The Spearman rank correlation coefficient were used to evaluate threshold effect performance and a *P* value < 0.05 indicates that the threshold effect may contribute to the heterogeneity. Using a bivariate random-effect model, the pooled sensitivity and specificity for [18F]FDG PET/CT and [18F]FDG PET/MRI were reported as estimates with 95% confidence intervals (CIs). The summary receiver operating characteristic curve and area under the curve (AUC) were generated by using the summary receiver operating characteristic (SROC) model. If the 95% confidence intervals of the two modalities did not overlap, it was considered that there was a statistically significant difference in performance. If the 95% confidence intervals of the AUC of the two modalities did not overlap, it was considered that there was a statistically significant difference in performance. Multilevel mixed-effects logistic regression was used to compare the summary paired sensitivity or specificity data, adding test type (PET/CT or PET/MRI) as covariate. Likelihood ratio tests were used to obtain the statistical differences between the sensitivities and specificities of the two tests type by fitting alternative models, adding or removing the covariate term from the model (20).

Heterogeneity among pooled studies was assessed using the I^2^ statistic. Homogeneity among the studies was considered to be low, moderate, or high when the I^2^ value was 25%, 50%, or 75%. For PET/CT, the meta-regression analysis and leave-one-out sensitivity analysis were conducted in the case of substantial heterogeneity (I^2^≥ 50%) to explore possible sources of heterogeneity. For PET/MRI, we did not conduct meta-regression due to the small number of included studies (less than 10), but we perform leave-one-out sensitivity analysis to find the source of heterogeneity. Deeks’ funnel plot tests were used to evaluate publication bias. All analyses were conducted with Stata 15.1 and Meta-DiSc 1.4. Statistical significance was defined as a *P* value less than 0.05.

### 2.2 Results

#### 2.2.1 Literature search and study selection

**Figure 1:**
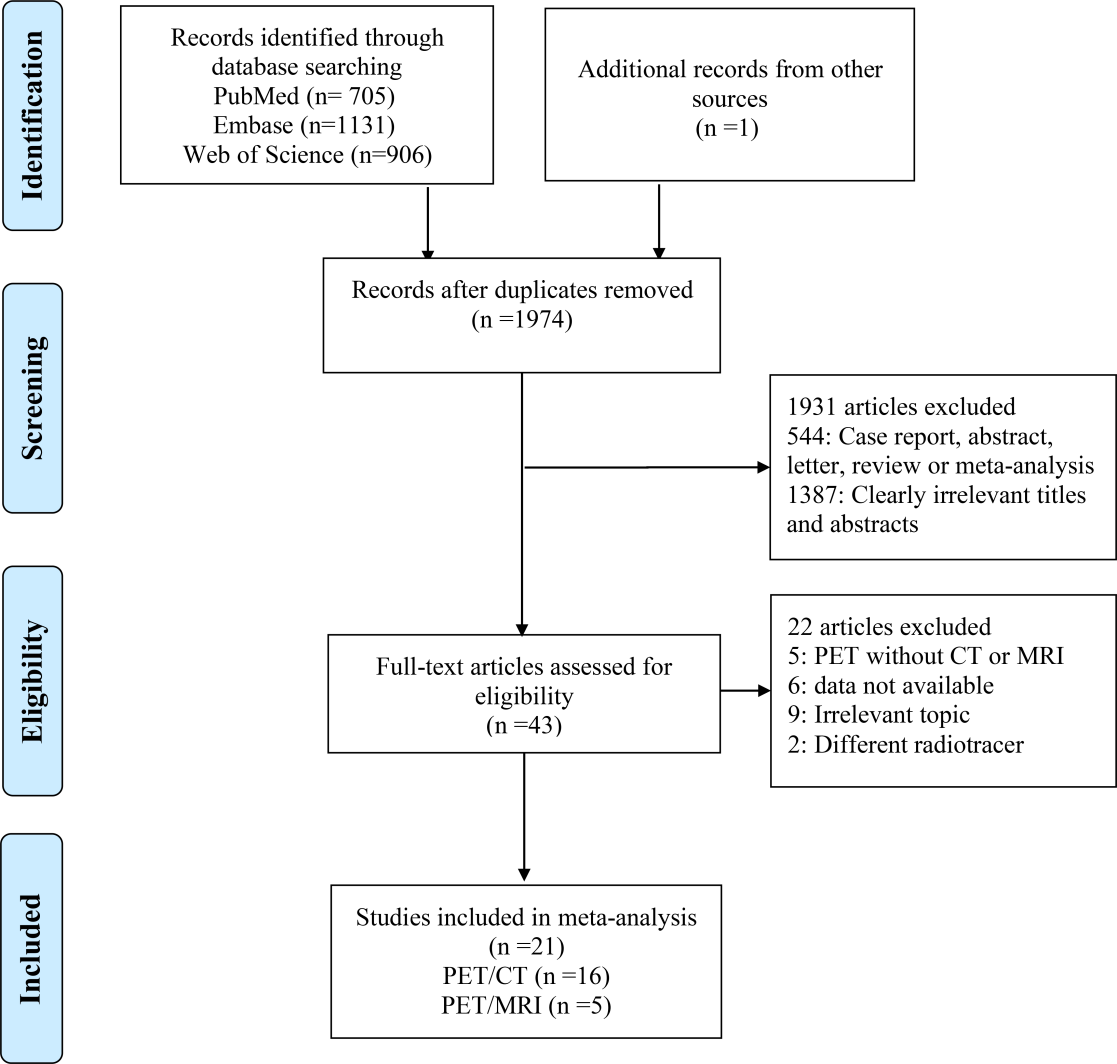
The PRISMA flow chart of study selection process.

There were a total of 2743 publications identified in the initial search, 1974 studies were identified after excluding 769 duplicated studies. Based on the title or abstract,1931 studies were excluded. In the remaining results, 9 articles were irrelevant, 2 used different radiotracers, 6 data not available, and 9 PET without CT or MRI. Finally, a total of 21 articles evaluating the diagnostic performance for colorectal liver metastasis including 16 articles for PET/CT (9, 10, 13–17, 21–29), and 5 articles for PET/MRI were included (17, 29–32). The PRISMA flow diagram of the study selection process is shown in **Figure 1**. A full list of all reviewed full-text articles was shown in **Supplementary Table 2**.

#### 2.2.2 Study description and quality assessment

**Figure 2:**
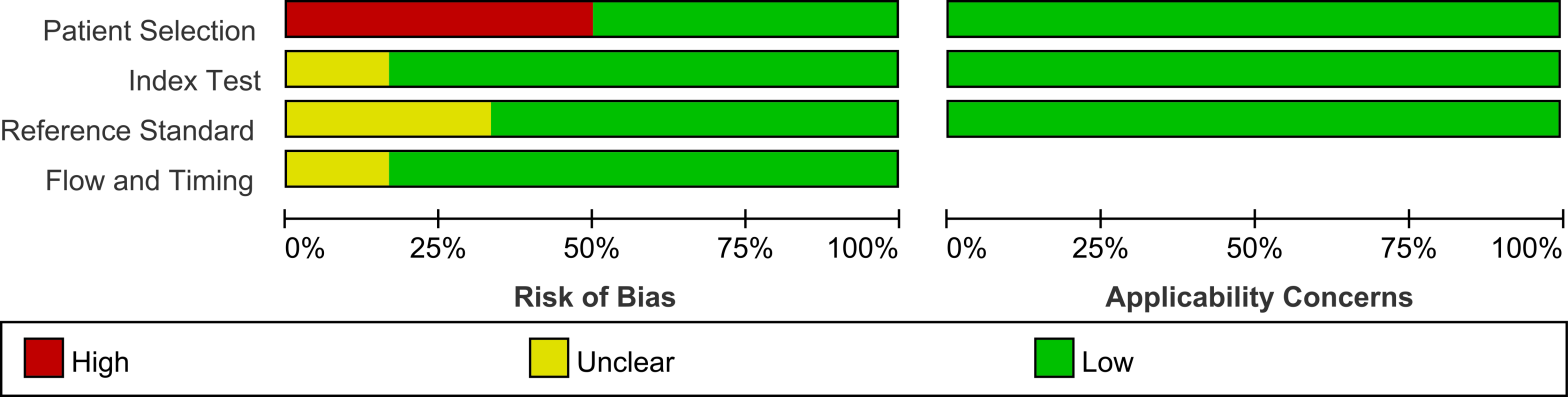
Graph of risk of bias and applicability of all eligible studies based on QUADAS-2 tool.

The study and patient characteristics from the 21 studies covering 1036 patients were listed in **Table 1** and **Table 2**. Technical aspects were displayed in **Table 3** and **Table 4**. What’ s more, an assessment on the quality of involved studies was carried out depending on the Quality Assessment of Diagnostic Accuracy Studies (QUADAS-2) tool. The quality assessment graph revealed high-risk bias concerns mainly concentrated on the field of patient selection (**Figure 2**), caused by that most of these studies did not include consecutive patients. Overall, the risk bias of the articles was considered satisfactory.

**Table 1:** Characteristics of the studies and patients for [18F]FDG PET/CT.

Author	Year	Types of imaging tests	Study characteristics					Patient characteristics				
			Country	Study design	Analysis	Reference standard	No. of patients		Clinical indication	Male/Female	Mean/Median age	Chemotherapy before PET (% of population)
Selzner et al. (14)	2004	PET/CT	Switzerland	Pro	PB	Pathology and/or follow-up imaging	76		Post-treatment	52/24	Mean = 63	62/76 (82%)
Rappeport et al. (16)	2007	PET/CT	Denmark	Pro	PB	Pathology and/or follow-up imaging	31		Initial staging Post-treatment	NA	NA	4/31 (13%)
Chua et al. (10)	2007	PET/CT	U.K.	Retro	PB	Pathology and/or follow-up imaging	75		Initial staging Post-treatment	NA	NA	NA
Lubezky et al. (21)	2007	PET/CT	Israel	Retro	LB	Pathology	48		Initial staging Post-treatment	25/23	Mean = 61.25	48/48 (100%)
Cantwell et al. (20)	2008	PET/CT	USA	Retro	LB	Pathology and/or follow-up imaging	33		Initial staging Post-treatment	22/11	Mean = 63	24/33 (73%)
Kong et al. (9)	2008	PET/CT	U.K.	Retro	LB	Pathology and/or follow-up imaging	65		Initial staging Post-treatment	42/23	Median = 65	NA
Mainenti et al. (22)	2010	PET/CT	Italy	Pro	PB	Pathology and/or follow-up imaging	34		Initial staging	20/14	Mean = 63	0/34 (0%)
Seo et al. (13)	2011	PET/CT	Korea	Retro	PB	Pathology and/or follow-up imaging	68		Initial staging Post-treatment	37/31	Mean = 63	26/68 (38%)
Ramos et al. (24)	2011	PET/CT	Spain	Pro	LB	Pathology and/or follow-up imaging	97		Initial staging Post-treatment	65/32	Mean = 63	27/97 (28%)
Garcia et al. (26)	2013	PET/CT	Spain	Pro	LB	Pathology and/or follow-up imaging	19		Post-treatment	13/6	Mean = 63	19/19 (100%)
Rojas-Llimpe et al. (25)	2014	PET/CT	Italy	Pro	LB	Pathology	51		Initial staging Post-treatment	31/20	Median = 65	27/51 (53%)
Schulz et al. (15)	2015	PET/CT	Norway	Pro	PB	Pathology and/or follow-up imaging	46		Initial staging Post-treatment	29/17	Mean = 67	10/46 (22%)
Brendle et al. (17)	2016	PET/CT	Germany	Retro	LB	Pathology and/or follow-up imaging	15		Initial staging Post-treatment	9/6	Mean = 45	12/15 (80%)
Mao et al. (23)	2020	PET/CT	China	Retro	PB	Pathology	108		Initial staging	77/31	Mean = 62.1	NO
Borello et al. (33)	2020	PET/CT	Italy	Retro	PB	Pathology and/or follow-up imaging	58		Initial staging Post-treatment	38/20	Mean = 65	46/58 (79%)
Yu et al. (27)	2021	PET/CT	China	Retro	PB	Pathology and/or follow-up imaging	27		Initial staging	19/8	Mean = 60.6	NO

PB, patient-based; LB, lesion-based; Pro, prospective; Retro, retrospective; NA, not available.

**Table 2:** Characteristics of the studies and patients for [18F]FDG PET/MRI.

Author	Year	Types of imaging tests	Study characteristics					Patient characteristics				
			Country	Study design	Analysis	Reference standard	No. of patients		Clinical indication	Male/Female	Mean/Median age	Chemotherapy before PET (% of population)
Brendle et al. (17)	2016	PET/MRI	Germany	Retro	LB	Pathology and/or follow-up imaging	15		Initial staging Post-treatment	9/6	Mean = 45	12/15(80%)
Yu et al. (27)	2021	PET/MRI	China	Retro	PB	Pathology and/or follow-up imaging	27		Initial staging	19/8	Mean = 60.6	NO
Lee et al. (29)	2015	PET/MRI	Korea	Pro	LB	Pathology	59		Initial staging Post-treatment	32/27	Mean = 58.3	6/59(10%)
Lee et al. (28)	2016	PET/MRI	Korea	Retro	PB	Pathology and/or follow-up imaging	55		Initial staging Post-treatment	42/13	Mean = 62.9	22/55(40%)
Yoon et al. (30)	2020	PET/MRI	Korea	Pro	LB	Pathology and/or follow-up imaging	71		Initial staging	43/28	Mean = 61	NO

PB, patient-based; LB, lesion-based; Pro, prospective; Retro, retrospective; NA, not available.

**Table 3:** Technical characteristics of the included studies for [18F]FDG PET/CT.

Author	Year	Types of imaging tests	Scanner Modality	Iodinated contrast medium	Ligand dose	Time from injection to acquisition	Image analysis	TP	FP	FN	TN	Total
Selzner et al. (14)	2004	PET/CT	Discovery LS, GE Medical Systems, Waukesha, WI	NO	370 MBq	45min	Visual: image interpretation was based on the identification of regions with increased FDG uptake on the PET images and the anatomic delineation of all FDG-avid lesions	61	1	6	8	76
Rappeport et al. (16)	2007	PET/CT	Discovery LS; GE Medical Systems, Milwaukee, Wisc., USA	YES	400 MBq	60min	Visual: image interpretation was based on criteria from daily practice and decide whether a lesion visible at PET, CT, or both was a benign or malignant lesion	26	0	2	3	31
Chua et al. (10)	2007	PET/CT	Discovery LS, GE, Michigan, USA	NO	370 MBq	NA	Visual: Lesions were analyzed qualitatively by visual assessment on multiplanar reconstructions and by examination of the maximum intensity tomographic data. Negative lesions were defined as those not associated with any focally increased FDG uptake above background levels	63	2	4	6	75
Lubezky et al. (21)	2007	PET/CT	Discovery LS; GE Medical Systems, Milwaukee, WI, USA	YES	370-666 MBq	60-120min	Visual: All suspected sites of metastatic disease showing an increased FDG uptake were recorded	48	4	50	20	122
Cantwell et al. (20)	2008	PET/CT	Biograph-16; Siemens Medical Solutions, Knoxville, Tenn	YES	555-740 MBq	18-36min	Visual: The diagnostic confidence of a reader in characterizing the lesion(s) was categorized into an ordinal scale of 0, no lesion or normal; 1, definitely benign; 2,probably benign; 3, possibly benign; 4, possibly malignant; 5,probably malignant; and 6, definitely malignant.	67	4	33	6	110
Kong et al. (9)	2008	PET/CT	Gemini, Philips	NO	400 MBq	60min	NA	155	0	10	6	171
Mainenti et al. (22)	2010	PET/CT	Discovery LS, GE Medical Systems, Milwaukee, USA	NO	370 MBq	60min	Quantitative: maximum-standardized-uptake-value(SUVmax)	6	1	0	27	34
Seo et al. (13)	2011	PET/CT	Discovery STE; GE Healthcare, Milwaukee, WI	YES	5.5 MBq/kg	60min	Visual: based on a 5-point confidence scale: 0, definitely not a metastasis; 1, probably not a metastasis; 2, possibly a metastasis; 3,probably a metastasis; 4, definitely a metastasis. A score of 0 was retrospectively assigned when an observer did not find a metastasis documented in any of the standard references. A lesion with a score of 3 or 4 was classified as positive.	57	2	4	5	68
Ramos et al. (24)	2011	PET/CT	Discovery ST scanner GE Healthcare, USA	NO	NA	NA	Visual: PET-CT findings were evaluated by a single experienced nuclear medicine physician with full knowledge of CT or MR findings to characterize visible lesions on CT and to detect new lesions unnoticed in the conventional study.	107	3	87	28	225
Garcia et al. (26)	2013	PET/CT	DSTE 16 s; GE Medical Systems	YES	370 MBq	60min	Quantitative: maximum-standardized-uptake-value(SUVmax)	109	0	6	5	120
Rojas-Llimpe et al. (25)	2014	PET/CT	GE, Discovery LS or GE Discovery STE	NO	370-555 MBq	60min	Visual: The PET/CT images were revised by two experienced nuclear medicine physicians unaware of the clinical data and the diagnosis was reached by consensus	85	2	56	18	161
Schulz et al. (15)	2015	PET/CT	Biograph mCT, Siemens AG, Erlangen, Germany	NO	4 MBq/kg	60min	Quantitative: maximum-standardized-uptake-value(SUVmax)	40	0	2	4	46
Brendle et al. (17)	2016	PET/CT	Biograph mCT Siemens Healthcare, Erlangen, Germany	NO	337 ± 59	62 ± 5min	Visual: Lesions were rated as a) malignant, b)benign, or, if they could not be assigned to the preceding, as c) equivocal, according to their appearance. Characterization of malignant lesions were performed according to standard clinical practice on a visual basis, quantitative parameters were not assessed.	7	1	15	14	37
Mao et al. (23)	2020	PET/CT	Discovery VCT scanner GE Healthcare, Milwaukee, Wisconsin, USA or uMI 510 scanner United Imaging Healthcare, Shanghai, China	NO	5.1 MBq/kg	164.6 ± 23.8min	Quantitative: maximum standardized uptake value(SUVmax) and mean standardized uptake value(SUVmean)	92	2	7	7	108
Borello et al. (33)	2020	PET/CT	Philips Ingenuity TF PET/CT scanner (Philips Medical Systems Inc; Cleveland OH)	NO	2.5 MBq/kg	50min	Quantitative: maximum standardized uptake value (SUVmax), mean standard uptake value (SUVmean), metabolic tumour volume (MTV) and total lesion glycolysis (TLG). A predefined threshold of 20% of the SUVmax was used to automatically generate volumes of interest.	51	0	6	1	58
Yu et al. (27)	2021	PET/CT	Biograph mCT (Siemens Healthcare, Erlangen, Germany)	NO	3.70-5.55MBq/kg	60min	Visual (1): When the metabolism of 18F-FDG of the lesion is higher than that of the surrounding liver tissue, the lesion is considered positive regardless of the density on CT (2); When the metabolism of the lesion is not significantly different from that of the surrounding liver tissue, the lesion with a slightly lower density on CT and not in accordance with the typical characteristics of cysts and hemangiomas is considered positive (3); When the metabolism of the lesion is lower than that of the surrounding liver tissue, the lesion is considered negative regardless of the density on CT (3) When the metabolism of the lesion is lower than the surrounding liver tissue, the lesion is considered negative regardless of the density on CT.	13	0	3	11	27

TP true positive; TN true negative; FP false positive; FN false positive; NA not available.

**Table 4:** Technical characteristics of the included studies for [18F]FDG PET/MRI.

Author	Year	Types ofimaging tests	Scanner Modality	Gadolinium contrast medium	Ligand dose	Time from injection to acquisition	Sequences	Image analysis	TP	FP	FN	TN	Total
Brendle et al. (17)	2016	PET/MRI	Biograph mMR Siemens Healthcare, Erlangen, Germany	NO	337 ± 59	120 ± 9min	T1WI with Dixon-based fat-water separation for attenuation correction; T2WI short-tau inversion recovery sequence; a navigator triggered axial fat-saturated T2-weighted turbo-spin echo; an axial fat-saturated T2WI TSE sequence of the pelvis; an echo-planar imaging sequence for DWI	Visual: Lesions were rated as a) malignant, b)benign, or, if they could not be assigned to the preceding, as c) equivocal, according to their appearance. Characterization of malignant lesions were performed according to standard clinical practice on a visual basis, quantitative parameters were not assessed.	16	3	6	12	37
Yu et al. (27)	2021	PET/MRI	Signa PET/MRI scanner GE Healthcare, USA	NO	3.70-5.55MBq/kg	NA	T2WI;T1WI;DWI	Visual: (1)When the metabolism of 18F-FDG of the lesion is higher than that of the surrounding liver tissue, the lesion is considered positive regardless of the signal on MRI; (2) When the metabolism of the lesion is not significantly different from that of the surrounding liver tissue, the lesion with low signal on T1WI, high signal on T2WI and restricted diffusion on DWI, and does not conform to the typical characteristics of cysts and hemangiomas is considered positive; (3) When the metabolism of the lesion is lower than that of the surrounding liver tissue, the lesion is considered negative regardless of the signal on MRI.	15	0	1	11	27
Lee et al. (29)	2015	PET/MRI	Biograph mMR (Siemens Healthcare, Erlangen, Germany)	NO	5.18 MBq/kg	75 ± 20 min	Two-point VIBE; Dixon whole-body DWI	Visual: Compared with the metabolism of normal liver parenchyma, hypermetabolic activity was considered a positive finding, and it matched on MRI images. Hepatic lesions with either hypometabolism, ambiguous metabolism, or isometabolism were considered benign findings or nonviable tumors. After chemotherapy of liver metastasis, detectable hepatic lesions with hypometabolism or isometabolism on PET/Dixon-VIBE/T1/T2 MRI were also considered nonviable tumors.	96	0	19	17	132
Lee et al. (28)	2016	PET/MRI	Biograph mMR (Siemens Healthcare, Erlangen, Germany)	YES	5.18 MBq	60min	Dixon-VIBE; T2WI	Visual: Each reader was asked to record the size and location of the lesions and to assess the probability of colorectal cancer liver metastases for each lesion by using the following five-point confidence scale: 1,definitely not colorectal cancer liver metastases; 2, probably not colorectal cancer liver metastases; 3, inde terminate; 4, probably colorectal cancer liver metastases; and 5, definitely colorectal cancer liver metastases. Prior to image interpretation, readers were instructed that scoring a lesion as 4 or 5 might be considered a positive diagnosis for colorectal cancer liver metastases.	41	0	7	7	55
Yoon et al. (30)	2020	PET/MRI	Biograph mMR (Siemens Healthcare, Erlangen, Germany)	YES	5.18 MBq/kg	60min	Two-point VIBE; Dixon whole-body DWI	Visual: FDG uptake on PET images was evaluated with visual assessment and compared with that in background liver, muscles, or contralateral lymph nodes.	10	0	1	60	71

TP, true positive; TN, true negative; FP, false positive; FN, false positive; NA, not available; VIBE, volumetric interpolated breath-hold examination; DWI, diffusion-weighted imaging; T1WI, T1-weighted imaging; T2WI, T2-weighted imaging.

#### 2.2.3 Diagnostic performance of [18F]FDG PET/CT and PET/MRI for colorectal liver metastasis

**Figure 3:**
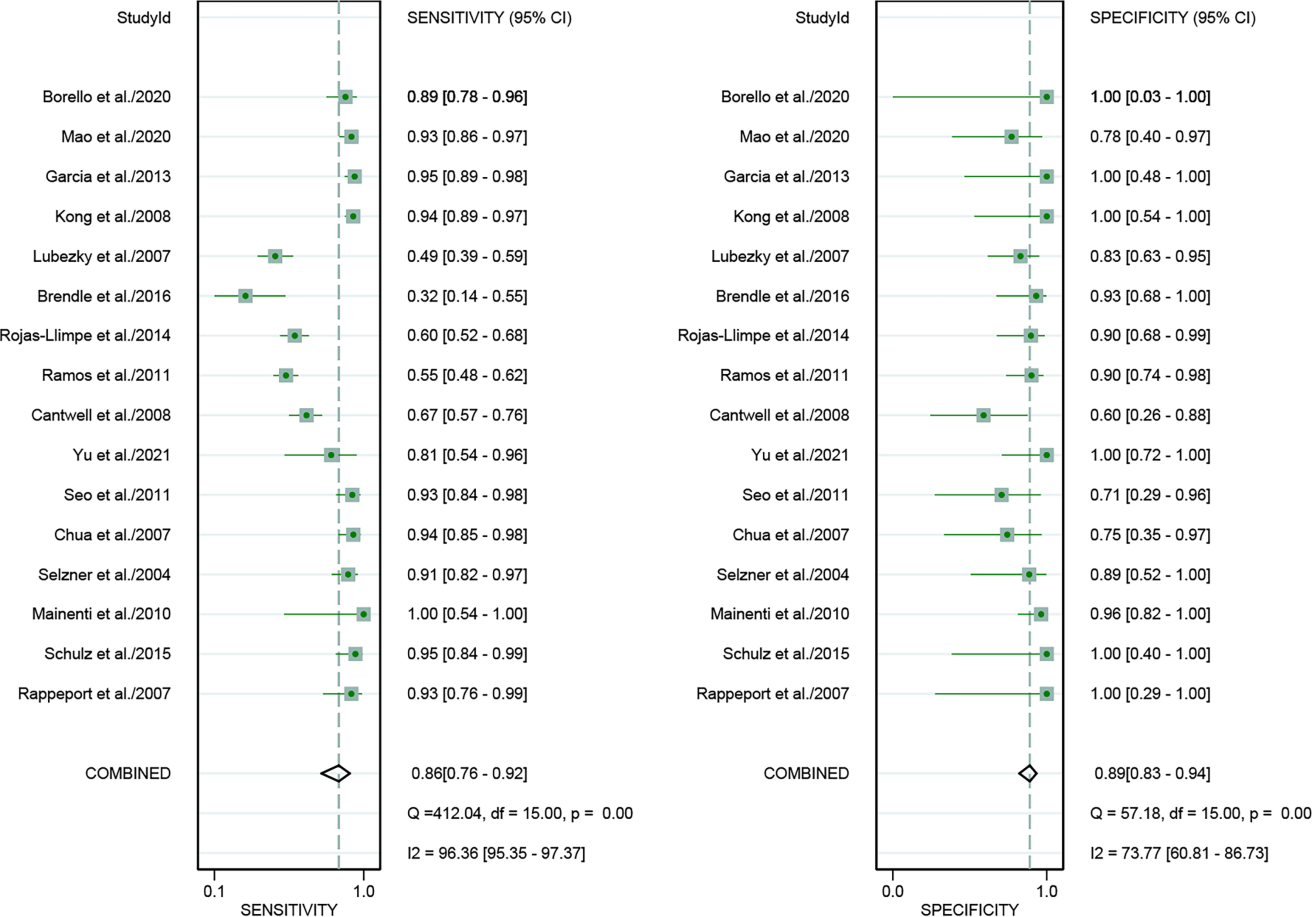
Forest plots of the combined [18F]FDG PET/CT sensitivity and specificity for colorectal liver metastasis. Squares denoted the sensitivity and specificity in each study, while horizontal bars indicated the 95% confidence interval.

**Figure 4:**
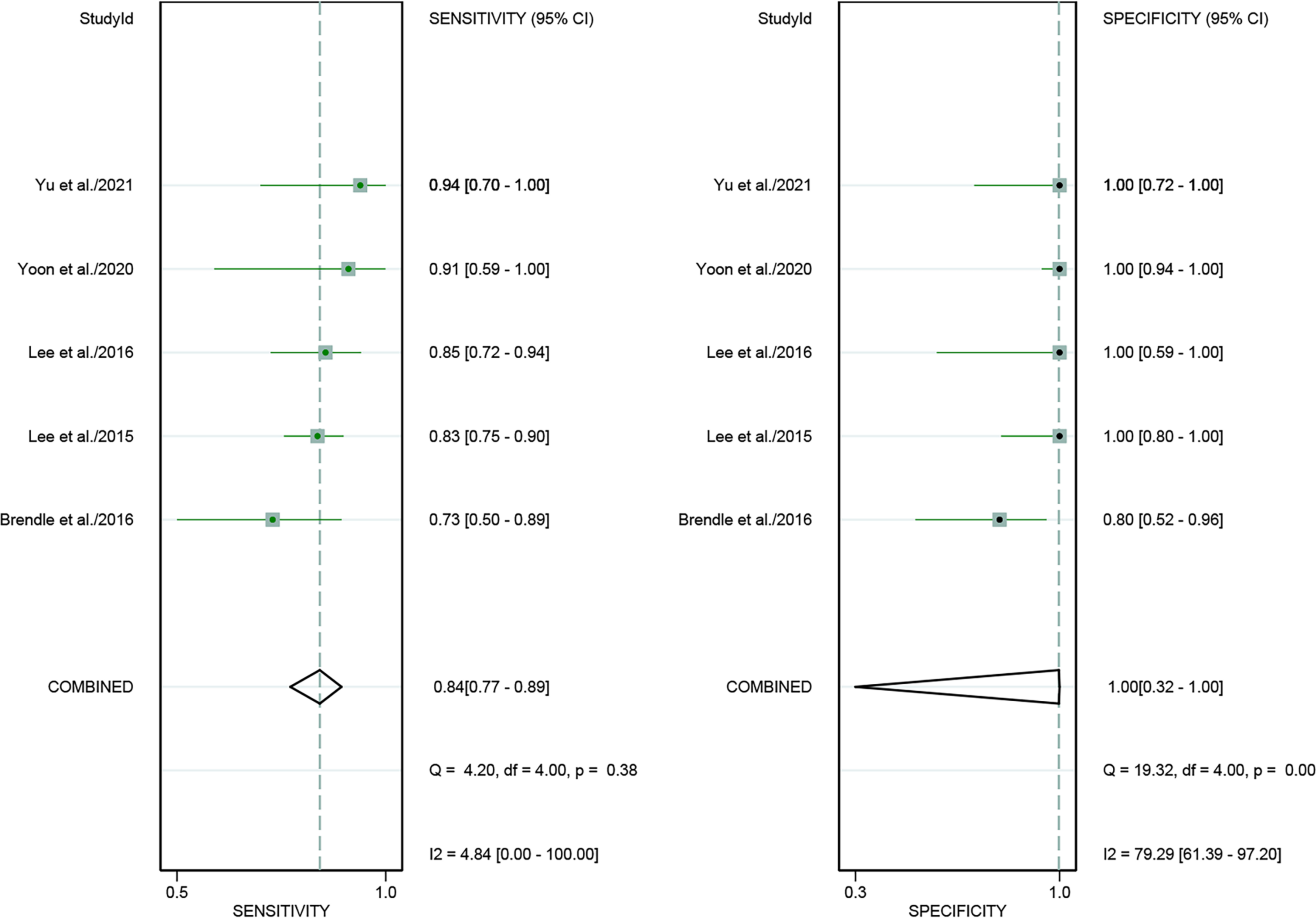
Forest plots of the combined [18F]FDG PET/MRI sensitivity and specificity for colorectal liver metastasis. Squares denoted the sensitivity and specificity in each study, while horizontal bars indicated the 95% confidence interval.

**Figure 5:**
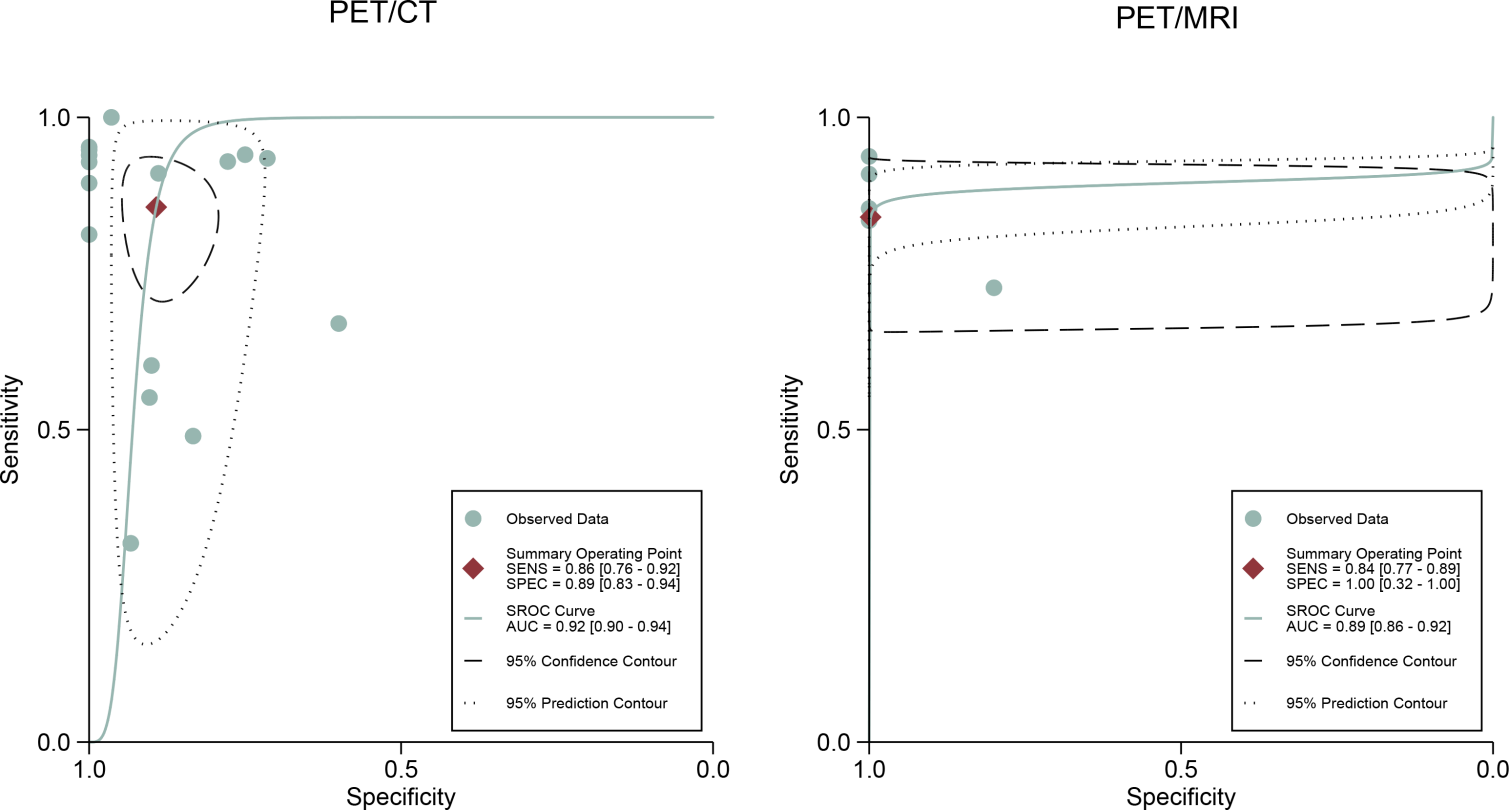
[18F]FDG PET/CT and [18F]FDG PET/MRI summary receiver operating characteristic (SROC) curves. The summary point is the optimal combination of sensitivity and specificity. The black dotted lines surrounding each summary point indicates the 95% confidence interval.

For [18F]FDG PET/CT, the results of the Spearman correlation coefficient demonstrated no threshold effect heterogeneity (Spearman correlation coefficient =0.074, *P*=0.786), similarly, [18F]FDG PET/MRI also showed no threshold effect heterogeneity (Spearman correlation coefficient =-0.500, *P*=0.391). The results of pooled sensitivity of [18F]FDG PET/CT for colorectal liver metastasis were 0.86 (95% CI, 0.76-0.92) and specificity were 0.89 (95% CI, 0.83-0.94) (**Figure 3**). The pooled sensitivity of [18F]FDG PET/MRI were 0.84 (95% CI, 0.77-0.89) and specificity were 1.00 (95%CI, 0.32-1.00) (**Figure 4**). The sensitivity of the two tests didn’t differ significantly (*P*= 0.58),and the specificity of the two tests also didn’t differ significantly (*P*= 0.27). **Figure 5** illustrated the SROC curve for [18F]FDG PET/CT and [18F]FDG PET/MRI, which exhibited an AUC of 0.92 (95%CI: 0.90-0.94) and 0.89 (95% CI: 0.86-0.92).

**Figure 6:**
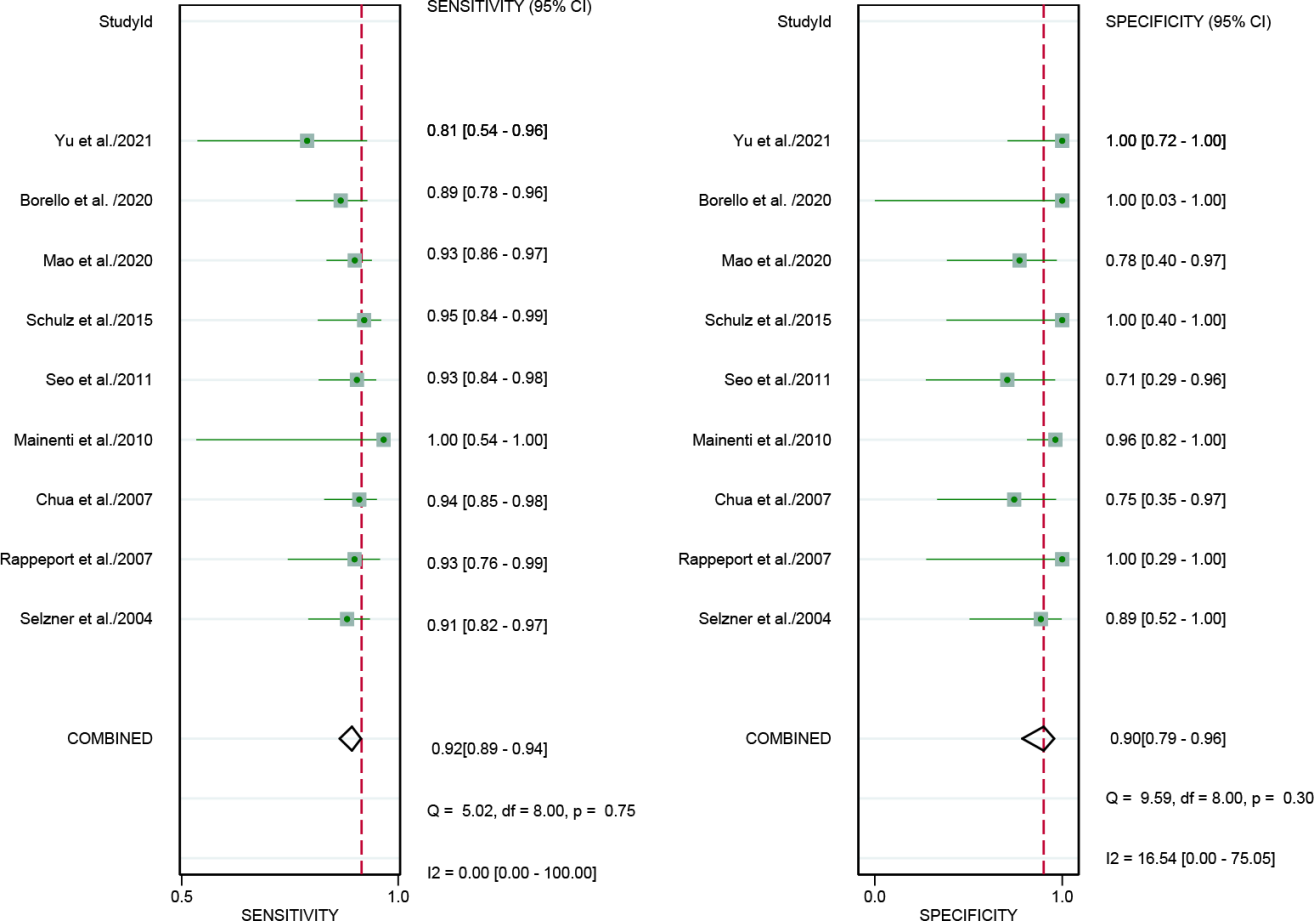
Forest plots of the combined [18F]FDG PET/CT sensitivity and specificity in patient-based analysis for colorectal liver metastasis. Squares denoted the sensitivity and specificity in each study, while horizontal bars indicated the 95% confidence interval.

In addition, we also performed a subgroup forest plot for PET/CT in patient-based analysis (**Figure 6**), the results of pooled sensitivity of [18F]FDG PET/CT in patient-based analysis for colorectal liver metastasis were 0.92 (95% CI, 0.82-0.97) and specificity were 0.89 (95% CI, 0.52-1.00).

#### 2.2.4 Heterogeneity analysis

**Figure 7:**
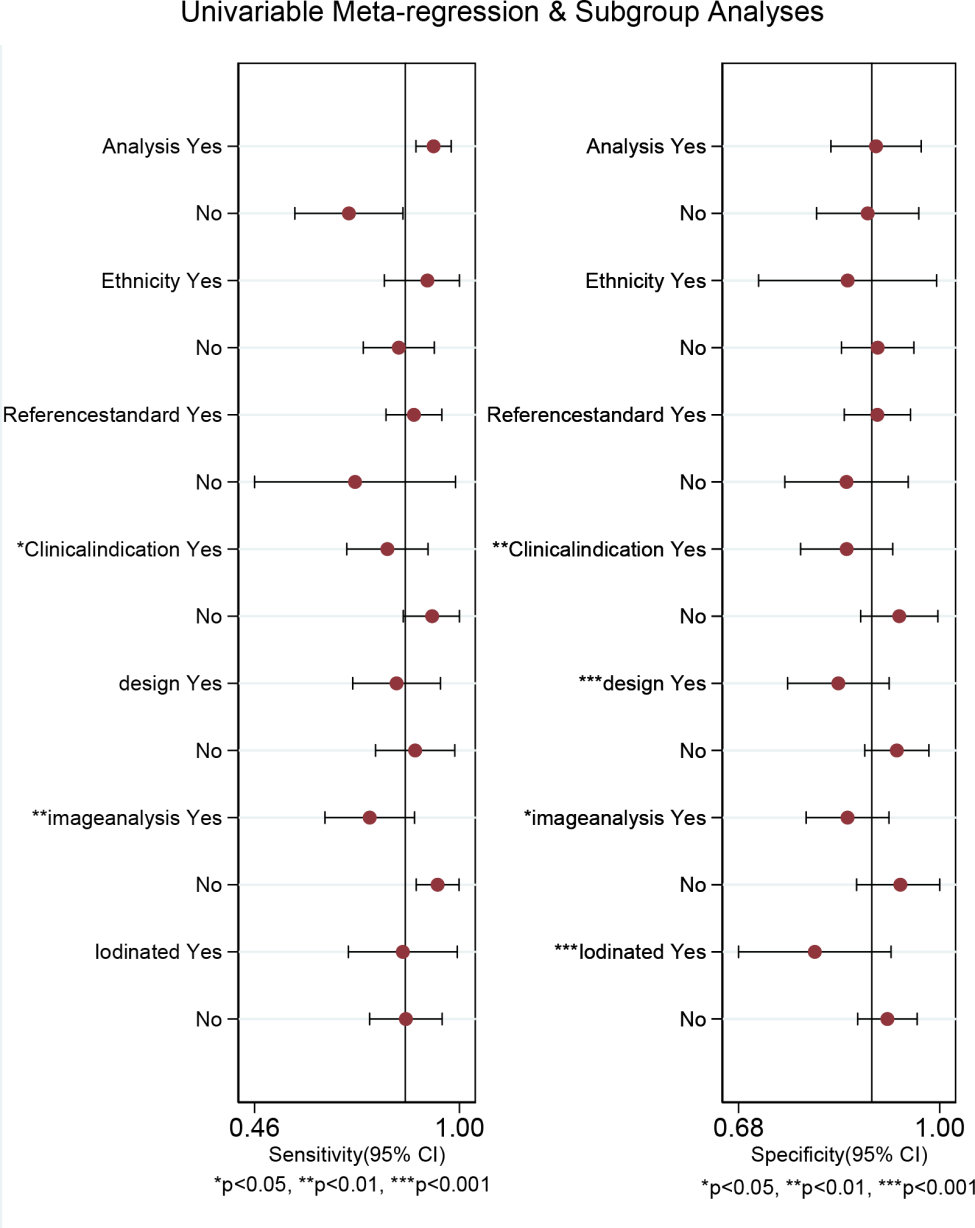
Multiple univariable meta-regression forest plot of [18F]FDG PET/CT for colorectal liver metastasis.

Regarding the pooled sensitivity and specificity of [18F]FDG PET/CT for colorectal liver metastasis, the I^2^ was 96.36%, 73.77%, respectively. In terms of the heterogeneity of [18F]FDG PET/MRI, the I^2^ were 79.29% and 4.84%. For [18F]FDG PET/CT, meta-regression analysis showed that image analysis(*P*<0.001 for sensitivity, *P*=0.02 for specificity), study design (*P*<0.001 for specificity), clinical indication (*P*=0.02 for sensitivity, *P*=0.01 for specificity), and iodinated contrast medium (*P*<0.001 for specificity) were the possible cause of heterogeneity (**Table 5** and **Figure 7**). For [18F]FDG PET/MRI, sensitivity analysis by excluding data from Brendle et al. demonstrated a combined specificity of 1.00(95% CI: 1.00–1.00), with no heterogeneity (I^2^ = 0%) (**Table 6**).

**Table 5:** Meta-regression analysis of 18F-FDG PET/CT for colorectal liver metastasis.

Covariate	Studies, n	Sensitivity (95%CI)	*P*-value	Specificity (95%CI)	*P*-value
Analysis			0.37		0.16
Patient-based	9	0.93 (0.88-0.98)		0.90 (0.83-0.97)	
Lesion-based	7	0.71 (0.56-0.85)		0.89 (0.81-0.97)	
Ethnicity			0.68		0.25
Asia	12	0.86 (0.77-0.95)		0.91 (0.85-0.96)	
Others	4	0.84 (0.68-1.00)		0.85 (0.75-0.95)	
Reference standard			0.46		0.19
Pathology	3	0.72 (0.46-0.99)		0.85 (0.76-0.95)	
Pathology and/or follow-up imaging	13	0.88 (0.81-0.95)		0.90 (0.85-0.95)	
Clinical indication			0.02		0.01
Initial staging and post-treatment	11	0.81 (0.70-0.92)		0.85 (0.78-0.93)	
Initial staging or post-treatment	5	0.93 (0.85-1.00)		0.94 (0.88-1.00)	
Study design			0.16		<0.001
Retrospective	9	0.83 (0.72-0.95)		0.84 (0.76-0.92)	
Prospective	7	0.88 (0.78-0.99)		0.93 (0.88-0.98)	
Image analysis			<0.001		0.02
Visual	10	0.76 (0.64-0.88)		0.85 (0.79-0.92)	
Quantitative	5	0.94 (0.89-1.00)		0.94 (0.87-1.00)	
Iodinated contrast medium			0.41		<0.001
Yes	5	0.85 (0.71-0.99)		0.80 (0.68-0.92)	
No	11	0.86 (0.76-0.95)		0.92 (0.87-0.96)	

**Table 6:** Sensitivity analysis of 18F-FDG PET/MRI for colorectal liver metastasis.

	Sensitivity (95%CI)	I^2^	Specificity (95%CI)	I^2^
Omitting Brendle et al. (17)	0.85 (0.85-0.85)	0%	1.00 (1.00-1.00)	0%
Omitting Lee et al. (2015) (29)	0.86 (0.73-0.93)	46.47%	0.99 (0.39-1.00)	81.51%
Omitting Lee et al. (2016) (28)	0.84 (0.74-0.91)	41.23%	1.00 (0.36-1.00)	83.49%
Omitting Yoon et al. (30)	0.83 (0.76-0.89)	6.63%	0.99 (0.37-1.00)	59.71%
Omitting Yu et al. (27)	0.83 (0.76-0.88)	0%	1.00 (0.37-1.00)	81.79%

#### 2.2.5 Publication bias

**Figure 8:**
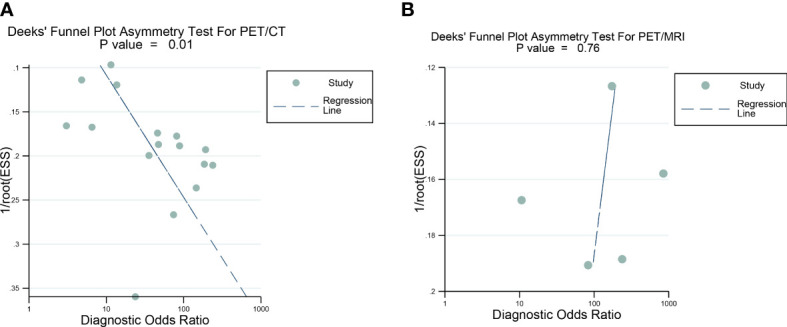
Deek’s funnel plot was used to evaluate the publication bias of [18F]FDG PET/CT and [18F]FDG PET/MRI. **(A)** Deek's funnel plot for PET/CT; **(B)** Deek's funnel plot for PET/CT. P<0.05 was considered significant.

Deeks’ funnel plot asymmetry test showed that there was a significant publication bias for [18F]FDG PET/CT (*P*=0.01), and no significant publication bias was observed for [18F]FDG PET/MRI (*P*=0.76) (**Figure 8**).

#### 2.3 Discussion

This is, to our knowledge, the first systematic review and meta-analysis comparing the diagnostic performance of [18F]FDG PET/CT and [18F]FDG PET/MRI for colorectal liver metastasis. The pooled sensitivity, specificity, and AUC of [18F]FDG PET/CT in were 0.86, 0.89, and 0.92. [18F]FDG PET/MRI were 0.84 and 1.00, and 0.89. PET/CT and PET/MRI seemed to have similar performance in detecting colorectal liver metastases because their 95% confidence intervals for AUC values were highly overlapping.

The staging or restaging of colorectal cancer’s local and distant metastases is crucial in assessing a patient’s survival and risk of recurrence, which was valuable for choosing the appropriate treatment strategy. Previously, three meta-analyses on PET or PET/CT for colorectal liver metastasis have been published. Niekel et al. conducted a meta-analysis that included only prospective trials (34). The authors reported that PET had a higher sensitivity on a per-patient basis (0.94), in comparison to both MRI (0.88) and CT (0.75). According to another meta-analysis conducted by Floriani et al., PET showed the highest sensitivity (0.94), followed by MRI (0.81), CT (0.75), and ultrasound for the diagnosis of liver metastases (6). A meta-analysis conducted by Maffione et al. indicated that PET is less sensitive (0.93) but more specific (0.93) than MRI and has an impact on the therapy of roughly one-fourth of patients (35). However, all of the previous meta-analyses didn’t mention hybrid PET/MRI system, which was an effective modality in detecting colorectal liver metastasis proven by recent studies. Thus, there was an urgent need to assess the diagnostic performance of PET/CT versus PET/MRI.

Our pooled sensitivity for PET/CT was inferior compared with the findings of both previously published meta-analyses (0.86 vs. 0.94 and 0.94). The specificity of PET/CT in our results were also inferior compared with the results of previous meta-analysis (0.89 vs. 0.94 and 0.93). This was due to our inclusion of studies that analyzed data from both patients and lesions. When we pooled sensitivity with only patient-based studies, the sensitivity (0.93) and specificity (0.90) were in line with the previous meta-analyses.

Even while [18F]FDG PET/CT and PET/MRI were increasingly being utilized to detect distant metastases in suspected cases of colorectal cancer, they were not the first-choice modality in the routine evaluation of CRC. However, Ruers et al. demonstrated the addition of PET over conventional imaging modalities, which indicated that the number of inefficient surgical procedures was dramatically decreased by include PET in the presurgical work-up (36). While [18F]FDG PET/CT has proven to be successful in a variety of clinical settings, [18F]FDG PET/MRI has not met with the same level of success. There were several reasons accounting for it, including expenses, logistical difficulties, and the original perception that PET/MRI was not superior to PET/CT in staging diagnostic ability (37), which was further confirmed in our study. Our results based on patient and lesion levels showed similar diagnostic performance between PET/CT and PET/MRI. When considering the high cost and diagnostic performance of PET/MRI, it was determined that the diagnostic value offered by PET/CT was effective enough to serve as a suitable alternative. In terms of the diagnostic performance, our findings may be applicable and timely for clinical decision-making. However, due to the small sample size of hybrid PET/MRI, further larger prospective studies focusing in the diagnostic performance of PET/MRI in colorectal liver metastasis are still needed to obtain a more robust result.

In terms of heterogeneity, there was high heterogeneity in [18F]FDG PET/CT (sensitivity and specificity) and [18F]FDG PET/MRI (specificity). We examined the sources of heterogeneity among the studies by performing meta-regression and sensitivity analysis. For [18F]FDG PET/CT, meta-regression analysis showed that image analysis, study design,clinical indication, and iodinated contrast medium were the possible cause of heterogeneity. For [18F]FDG PET/MRI, we got an acceptable heterogeneity (I^2 =^ 0%) by excluding data from Brendle et al., which could be explained by different previous treatment and cut-off thresholds. Nevertheless, there may be further causes, such as differences in patients, technique, and study design. It yielded a same specificity (1.00) when we omitting the study by Brendle et al., which further proved the robustness of the results.

The limitations of our meta-analysis should also be mentioned. First, only five studies offered sufficient information that evaluated the diagnostic performance of the hybrid PET/MRI system for the staging or restaging of liver metastases in CRC patients, which leads to small sample size. This was because hybrid PET/MRI were introduced in the recent years and still lack of well-designed trials. Second, Due to the limited number of research that satisfied the inclusion criteria, we included either patient-based or lesion-based analysis studies, which could increase potential bias. Third, most of the reference standard for diagnosis colorectal liver metastasis was pathology and follow-up imaging, pathological results are not obtained for all patients in the included studies. These results therefore need to be interpreted with caution.

### 2.4 Conclusion

[18F]FDG PET/CT shows similar performance compared to [18F]FDG PET/MRI in detecting colorectal liver metastasis. However, pathological results were not obtained for all patients in the included studies and PET/MRI results were derived from studies with small sample sizes. There is a need for additional, larger prospective studies on this issue.

## Data availability statement

The original contributions presented in the study are included in the article/**Supplementary Material**. Further inquiries can be directed to the corresponding author.

## Author contributions

ZM and XZ conceived and designed the study, which were proofed by ZM. XZ and XL collected and analyzed the data. ZM wrote the manuscript. All authors contributed to the article and approved the submitted version.
