# Supplementary material for: [18F]FDG PET/CT versus [18F]FDG PET/MRI for the diagnosis of colorectal liver metastasis: A systematic review and meta-analysis
Source: Front Oncol. 2023 Feb 13;13:1114059. doi: 10.3389/fonc.2023.1114059 (PMC9969139; doi:10.3389/fonc.2023.1114059)
Supplement: Supplementary file 1 [file DataSheet_1.docx]

Supplementary Table 1 PRISMA checklist.

| **Section/topic** | **#** | **Checklist item** | **Reported on page #** |  |  |
| --- | --- | --- | --- | --- | --- |
| **TITLE** | | |  |  |  |
| Title | 1 | Identify the report as a systematic review, meta-analysis, or both. | Page 1 |  |  |
| **ABSTRACT** | | |  |  |  |
| Structured summary | 2 | Provide a structured summary including, as applicable: background; objectives; data sources; study eligibility criteria, participants, and interventions; study appraisal and synthesis methods; results; limitations; conclusions and implications of key findings; systematic review registration number. | Page1-2 |  |  |
| **INTRODUCTION** | | |  |  |  |
| Rationale | 3 | Describe the rationale for the review in the context of what is already known. | Page2 |  |  |
| Objectives | 4 | Provide an explicit statement of questions being addressed with reference to participants, interventions, comparisons, outcomes, and study design (PICOS). | Page2 |  |  |
| **METHODS** | | |  |  |  |
| Protocol and registration | 5 | Indicate if a review protocol exists, if and where it can be accessed (e.g., Web address), and, if available, provide registration information including registration number. | Not available |  |  |
| Eligibility criteria | 6 | Specify study characteristics (e.g., PICOS, length of follow-up) and report characteristics (e.g., years considered, language, publication status) used as criteria for eligibility, giving rationale. | Page 3 |  |  |
| Information sources | 7 | Describe all information sources (e.g., databases with dates of coverage, contact with study authors to identify additional studies) in the search and date last searched. | Page 3 |  |  |
| Search | 8 | Present full electronic search strategy for at least one database, including any limits used, such that it could be repeated. | Page 3 |  |  |
| Study selection | 9 | State the process for selecting studies (i.e., screening, eligibility, included in systematic review, and, if applicable, included in the meta-analysis). | Page 3 |  |  |
| Data collection process | 10 | Describe method of data extraction from reports (e.g., piloted forms, independently, in duplicate) and any processes for obtaining and confirming data from investigators. | Page 4 |  |  |
| Data items | 11 | List and define all variables for which data were sought (e.g., PICOS, funding sources) and any assumptions and simplifications made. | Page 3-4 |  |  |
| Risk of bias in individual studies | 12 | Describe methods used for assessing risk of bias of individual studies (including specification of whether this was done at the study or outcome level), and how this information is to be used in any data synthesis. | Page 3 |  |  |
| Summary measures | 13 | State the principal summary measures (e.g., risk ratio, difference in means). | Page 3-4 |  |  |
| Synthesis of results | 14 | Describe the methods of handling data and combining results of studies, if done, including measures of consistency (e.g., I^2^) for each meta-analysis. | Page 3-4 |  |  |

| Risk of bias across studies | 15 | Specify any assessment of risk of bias that may affect the cumulative evidence (e.g., publication bias, selective reporting within studies). | Page 4 |  |  |
| --- | --- | --- | --- | --- | --- |
| Additional analyses | 16 | Describe methods of additional analyses (e.g., sensitivity or subgroup analyses, meta-regression), if done, indicating which were pre-specified. | Not available |  |  |
| **RESULTS** | | |  |  |  |
| Study selection | 17 | Give numbers of studies screened, assessed for eligibility, and included in the review, with reasons for exclusions at each stage, ideally with a flow diagram. | Page 4 |  |  |
| Study characteristics | 18 | For each study, present characteristics for which data were extracted (e.g., study size, PICOS, follow-up period) and provide the citations. | Page 4-5 |  |  |
| Risk of bias within studies | 19 | Present data on risk of bias of each study and, if available, any outcome level assessment (see item 12). | Page 4-5 |  |  |
| Results of individual studies | 20 | For all outcomes considered (benefits or harms), present, for each study: (a) simple summary data for each intervention group (b) effect estimates and confidence intervals, ideally with a forest plot. | Page 4-5 |  |  |
| Synthesis of results | 21 | Present results of each meta-analysis done, including confidence intervals and measures of consistency. | Page 5 |  |  |
| Risk of bias across studies | 22 | Present results of any assessment of risk of bias across studies (see Item 15). | Page 5 |  |  |
| Additional analysis | 23 | Give results of additional analyses, if done (e.g., sensitivity or subgroup analyses, meta-regression [see Item 16]). | Not available |  |  |
| **DISCUSSION** | | |  |  |  |
| Summary of evidence | 24 | Summarize the main findings including the strength of evidence for each main outcome; consider their relevance to key groups (e.g., healthcare providers, users, and policy makers). | Page 6 |  |  |
| Limitations | 25 | Discuss limitations at study and outcome level (e.g., risk of bias), and at review-level (e.g., incomplete retrieval of identified research, reporting bias). | Page 7 |  |  |
| Conclusions | 26 | Provide a general interpretation of the results in the context of other evidence, and implications for future research. | Page 7 |  |  |
| **FUNDING** | | |  |  |  |
| Funding | 27 | Describe sources of funding for the systematic review and other support (e.g., supply of data); role of funders for the systematic review. | Not available |  |  |

Supplementary Table 2 A full list of all reviewed full-text articles.

| Author | Study included in the meta-analysis | PET without CT or MRI | Data not available to calculate | Irrelevant topic | Different radiotracer |
| --- | --- | --- | --- | --- | --- |
| Selzner et al.(1) | √ |  |  |  |  |
| Rappeport et al.(2) | √ |  |  |  |  |
| Chua et al.(3) | √ |  |  |  |  |
| Lubezky et al.(4) | √ |  |  |  |  |
| Cantwell et al.(5) | √ |  |  |  |  |
| Kong et al.(6) | √ |  |  |  |  |
| Mainenti et al.(7) | √ |  |  |  |  |
| Seo et al.(8) | √ |  |  |  |  |
| Ramos et al.(9) | √ |  |  |  |  |
| Garcia et al.(10) | √ |  |  |  |  |
| Rojas-Llimpe et al.(11) | √ |  |  |  |  |
| Schulz et al.(12) | √ |  |  |  |  |
| Brendle et al.(13) | √ |  |  |  |  |
| Mao et al.(14) | √ |  |  |  |  |
| Borello et al.(15) | √ |  |  |  |  |
| Yu et al.(16) | √ |  |  |  |  |
| Lee et al.(17) | √ |  |  |  |  |
| Lee et al.(18) | √ |  |  |  |  |
| Yoon et al.(19) | √ |  |  |  |  |
| Ruers et al.(20) |  | √ |  |  |  |
| Carnaghi et al.(21) |  | √ |  |  |  |
| Ravikumar et al.(22) |  | √ |  |  |  |
| Akhurst et al.(23) |  | √ |  |  |  |
| Lai et al.(24) |  | √ |  |  |  |
| Zangheri et al.(25) |  |  | √ |  |  |
| Chiu et al.(26) |  |  | √ |  |  |
| Choi et al.(27) |  |  | √ |  |  |
| Falconer et al.(28) |  |  | √ |  |  |
| Gasparini et al.(29) |  |  | √ |  |  |
| Hotta et al.(30) |  |  | √ |  |  |
| Lee et al.(31) |  |  |  | √ |  |
| Mao et al.(32) |  |  |  | √ |  |
| Nishioka et al.(33) |  |  |  | √ |  |
| Shim et al.(34) |  |  |  | √ |  |
| Tam et al.(35) |  |  |  | √ |  |
| Soydal et al.(36) |  |  |  | √ |  |
| Abbadi et al.(37) |  |  |  | √ |  |
| Moulton et al.(38) |  |  |  | √ |  |
| Nielsen et al.(39) |  |  |  | √ |  |
| Cuda et al.(40) |  |  |  |  | √ |
| Albert et al.(41) |  |  |  |  | √ |

Reference:

1. Selzner MK, Hany TF, Wildbrett P, McCormack L, Kadry Z, Clavien PA. Does the novel PET/CT imaging modality impact on the treatment of patients with metastatic colorectal cancer of the liver? ANNALS OF SURGERY (2004) 240(6):1027-36. doi: 10.1097/01.sla.0000146145.69835.c5

2. Rappeport ED, Loft A, Berthelsen AK, von der Recke P, Larsen PN, Mogensen AM, et al. Contrast-enhanced FDG-PET/CT vs. SPIO-enhanced MRI vs. FDG-PET vs. CT in patients with liver metastases from colorectal cancer: A prospective study with intraoperative confirmation. ACTA RADIOLOGICA (2007) 48(4):369-78. doi: 10.1080/02841850701294560

3. Chua SC, Groves AM, Kayani I, Menezes L, Gacinovic S, Du Y, et al. The impact of (18)F-FDG PET/CT in patients with liver metastases. EUROPEAN JOURNAL OF NUCLEAR MEDICINE AND MOLECULAR IMAGING (2007) 34(12):1906-14. doi: 10.1007/s00259-007-0518-y

4. Lubezky N, Metser U, Geva R, Nakache R, Shmueli E, Klausner JM, et al. The role and limitations of 18-fluoro-2-deoxy-D-glucose positron emission tomography (FDG-PET) scan and computerized tomography (CT) in restaging patients with hepatic colorectal metastases following neoadjuvant chemotherapy: Comparison with operative and pathological findings. JOURNAL OF GASTROINTESTINAL SURGERY (2007) 11(4):472-8. doi: 10.1007/s11605-006-0032-8

5. Cantwell CP, Setty BN, Holalkere N, Sahani DV, Fischman AJ, Blake MA. Liver Lesion Detection and Characterization in Patients With Colorectal Cancer: A Comparison of Low Radiation Dose Non-enhanced PET/CT, Contrast-enhanced PET/CT, and Liver MRI. JOURNAL OF COMPUTER ASSISTED TOMOGRAPHY (2008) 32(5):738-44. doi: 10.1097/RCT.0b013e3181591d33

6. Kong G, Jackson C, Koh DM, Lewington V, Sharma B, Brown G, et al. The use of 18F-FDG PET/CT in colorectal liver metastases--comparison with CT and liver MRI. Eur J Nucl Med Mol Imaging (2008) 35(7):1323-9. Epub 20080318. doi: 10.1007/s00259-008-0743-z

7. Mainenti PP, Mancini M, Mainolfi C, Camera L, Maurea S, Manchia A, et al. Detection of colo-rectal liver metastases: prospective comparison of contrast enhanced US, multidetector CT, PET/CT, and 1.5 Tesla MR with extracellular and reticulo-endothelial cell specific contrast agents. ABDOMINAL IMAGING (2010) 35(5):511-21. doi: 10.1007/s00261-009-9555-2

8. Seo HJ, Kim MJ, Lee JD, Chung WS, Kim YE. Gadoxetate Disodium-Enhanced Magnetic Resonance Imaging Versus Contrast-Enhanced F-18-Fluorodeoxyglucose Positron Emission Tomography/Computed Tomography for the Detection of Colorectal Liver Metastases. INVESTIGATIVE RADIOLOGY (2011) 46(9):548-55. doi: 10.1097/RLI.0b013e31821a2163

9. Ramos E, Valls C, Martinez L, Llado L, Torras J, Ruiz S, et al. Preoperative Staging of Patients with Liver Metastases of Colorectal Carcinoma. Does PET/CT Really Add Something to Multidetector CT? ANNALS OF SURGICAL ONCOLOGY (2011) 18(9):2654-61. doi: 10.1245/s10434-011-1670-y

10. Vicente AMG, Ferreras ED, Perez VS, Garcia VMP, Guzman JCV, Aragon FJ, et al. Response assessment of colorectal liver metastases with contrast enhanced CT/18F-FDG PET. EUROPEAN JOURNAL OF RADIOLOGY (2013) 82(6):E255-E61. doi: 10.1016/j.ejrad.2012.12.029

11. Rojas Llimpe FL, Di Fabio F, Ercolani G, Giampalma E, Cappelli A, Serra C, et al. Imaging in resectable colorectal liver metastasis patients with or without preoperative chemotherapy: results of the PROMETEO-01 study. Br J Cancer (2014) 111(4):667-73. Epub 20140701. doi: 10.1038/bjc.2014.351

12. Schulz A, Viktil E, Godt JC, Johansen CK, Dormagen JB, Holtedahl JE, et al. Diagnostic performance of CT, MRI and PET/CT in patients with suspected colorectal liver metastases: the superiority of MRI. ACTA RADIOLOGICA (2016) 57(9):1040-8. doi: 10.1177/0284185115617349

13. Brendle C, Schwenzer NF, Rempp H, Schmidt H, Pfannenberg C, la Fougere C, et al. Assessment of metastatic colorectal cancer with hybrid imaging: comparison of reading performance using different combinations of anatomical and functional imaging techniques in PET/MRI and PET/CT in a short case series. EUROPEAN JOURNAL OF NUCLEAR MEDICINE AND MOLECULAR IMAGING (2016) 43(1):123-32. doi: 10.1007/s00259-015-3137-z

14. Mao W, Zhou J, Qiu L, Yin H, Tan H, Shi H. The added value of dual-time-point 18F-FDG PET/CT imaging in the diagnosis of colorectal cancer liver metastases. Abdom Radiol (NY) (2020) 45(4):1075-81. doi: 10.1007/s00261-019-02396-3

15. Borello A, Russolillo N, Lo Tesoriere R, Langella S, Guerra M, Ferrero A. Diagnostic performance of the FDG-PET/CT in patients with resected mucinous colorectal liver metastases. SURGEON-JOURNAL OF THE ROYAL COLLEGES OF SURGEONS OF EDINBURGH AND IRELAND (2021) 19(5):E140-E5. doi: 10.1016/j.surge.2020.09.004

16. Yu Jun ZY, Ren Dongdong, Chen Cong, Li Yang, Ren Chunling, Cai Jun. Comparative analysis of 18F-FDG PET/CT and PET/MRI in the diagnosis of colorectal cancer liver metastases. Int J Radiat Med Nucl Med (2021) 45(2). doi: 10.3760/cma.j.cn121381−202003009−00013

17. Lee DH, Lee JM, Hur BY, Joo I, Yi NJ, Suh KS, et al. Colorectal Cancer Liver Metastases: Diagnostic Performance and Prognostic Value of PET/MR Imaging. RADIOLOGY (2016) 280(3):782-92. doi: 10.1148/radiol.2016151975

18. Lee SJ, Seo HJ, Kang KW, Jeong SY, Yi NJ, Lee JM, et al. Clinical Performance of Whole-Body 18F-FDG PET/Dixon-VIBE, T1-Weighted, and T2-Weighted MRI Protocol in Colorectal Cancer. CLINICAL NUCLEAR MEDICINE (2015) 40(8):E392-E8. doi: 10.1097/RLU.0000000000000812

19. Yoon JH, Lee JM, Chang W, Kang HJ, Bandos A, Lim HJ, et al. Initial M Staging of Rectal Cancer: FDG PET/MRI with a Hepatocyte-specific Contrast Agent versus Contrast-enhanced CT. RADIOLOGY (2020) 294(2):310-9. doi: 10.1148/radiol.2019190794

20. Ruers TJ, Wiering B, van der Sijp JR, Roumen RM, de Jong KP, Comans EF, et al. Improved selection of patients for hepatic surgery of colorectal liver metastases with (18)F-FDG PET: a randomized study. J Nucl Med (2009) 50(7):1036-41. Epub 20090612. doi: 10.2967/jnumed.109.063040

21. Carnaghi C, Tronconi MC, Rimassa L, Tondulli L, Zuradelli M, Rodari M, et al. Utility of 18F-FDG PET and contrast-enhanced CT scan in the assessment of residual liver metastasis from colorectal cancer following adjuvant chemotherapy. Nucl Med Rev Cent East Eur (2007) 10(1):12-5.

22. Ravikumar TS, Jones M, Serrano M, Kaleya R, Valdivia A, Milstein DM. The role of PET scanning in radiofrequency ablation of liver metastasis from colorectal cancer. CANCER JOURNAL (2000) 6:S330-S43.

23. Akhurst T, Gonen M, Baser RE, Schwartz LH, Tuorto S, Brody LA, et al. Prospective evaluation of F-18-FDG positron emission tomography in the preoperative staging of patients with hepatic colorectal metastases. HEPATOBILIARY SURGERY AND NUTRITION. doi: 10.21037/hbsn-19-357

24. Abdel-Nabi H, Doerr RJ, Lamonica DM, Cronin VR, Galantowicz PJ, Carbone GM, et al. Staging of primary colorectal carcinomas with fluorine-18 fluorodeoxyglucose whole-body PET: correlation with histopathologic and CT findings. Radiology (1998) 206(3):755-60. doi: 10.1148/radiology.206.3.9494497

25. Zangheri B, Sollini M, Calabrese L, Gabanelli S, Gasparini M. Evaluation of dual-time-point F-18-FDG PET/CT in detection of liver metastasis. Clinical and Translational Imaging (2017) 5:S51-S2. doi: 10.1007/s40336-017-0227-x

26. Chiu KWH, Lam KO, An H, Cheung GTC, Lau JKS, Choy TS, et al. Long-term outcomes and recurrence pattern of 18F-FDG PET-CT complete metabolic response in the first-line treatment of metastatic colorectal cancer: a lesion-based and patient-based analysis. BMC CANCER (2018) 18. doi: 10.1186/s12885-018-4687-9

27. Choi EK, Oh JK, Chung YA. Prognostic value of staging FDG PET/CT in patients with metastatic colorectal cancer. Annals of Oncology (2018) 29. doi: 10.1093/annonc/mdy431.048

28. Falconer R, Connor S, Balasingam A, Eglinton T. Does positron emission tomography/computed tomography change management in colorectal cancer? ANZ JOURNAL OF SURGERY (2018) 88(4):E248-E51. doi: 10.1111/ans.13798

29. Gasparini M, Zangheri B, Calabrese L, Gabanelli S, Bestetti A. Diagnostic impact of dual-time-point 18F-FDG PET/CT in detection of liver metastasis. European Journal of Nuclear Medicine and Molecular Imaging (2018) 45:S163-S4. doi: 10.1007/s00259-018-4148-3

30. Hotta M, Minamimoto R, Yano H, Gohda Y, Shuno Y. Diagnostic performance of F-18-FDG PET/CT using point spread function reconstruction on initial staging of rectal cancer: a comparison study with conventional PET/CT and pelvic MRI. CANCER IMAGING (2018) 18. doi: 10.1186/s40644-018-0137-9

31. Lee JW, Baek MJ, Ahn TS, Lee SM. Fluorine-18-fluorodeoxyglucose uptake of bone marrow on PET/CT can predict prognosis in patients with colorectal cancer after curative surgical resection. Eur J Gastroenterol Hepatol (2018) 30(2):187-94. doi: 10.1097/meg.0000000000001018

32. Mao WJ, Zhou J, Zhang H, Shi HC. Relationship between KRAS Mutations and dual time point 18F-FDG PET/CT imaging in colorectal cancer liver metastases. JOURNAL OF NUCLEAR MEDICINE (2018) 59.

33. Nishioka Y, Yoshioka R, Gonoi W, Sugawara T, Yoshida S, Hashimoto M, et al. Fluorine-18-fluorodeoxyglucose positron emission tomography as an objective substitute for CT morphologic response criteria in patients undergoing chemotherapy for colorectal liver metastases. ABDOMINAL RADIOLOGY (2018) 43(5):1152-8. doi: 10.1007/s00261-017-1287-0

34. Shim JR, Lee SD, Han SS, Lee SJ, Lee DE, Kim SK, et al. Prognostic significance of F-18-FDG PET/CT in patients with colorectal cancer liver metastases after hepatectomy. EJSO (2018) 44(5):670-6. doi: 10.1016/j.ejso.2018.01.243

35. Tam H, Cook G, Koh DM, Chau I, Cunningham D, Chua S. Prognostic value of pretreatment 18F-FDG-PET/CT in colorectal liver metastasis. JOURNAL OF NUCLEAR MEDICINE (2010) 51.

36. Soydal C, Kucuk ON, Gecim EI, Bilgic S, Elhan AH. The prognostic value of quantitative parameters of 18F-FDG PET/CT in the evaluation of response to internal radiation therapy with yttrium-90 in patients with liver metastases of colorectal cancer. Nucl Med Commun (2013) 34(5):501-6. doi: 10.1097/MNM.0b013e32835f9427

37. Abbadi RA, Sadat U, Jah A, Praseedom RK, Jamieson NV, Cheow HK, et al. Improved long-term survival after resection of colorectal liver metastases following staging with FDG positron emission tomography. J Surg Oncol (2014) 110(3):313-9. Epub 20140416. doi: 10.1002/jso.23623

38. Moulton CA, Gu CS, Law CH, Tandan VR, Hart R, Quan D, et al. Effect of PET Before Liver Resection on Surgical Management for Colorectal Adenocarcinoma Metastases A Randomized Clinical Trial. JAMA-JOURNAL OF THE AMERICAN MEDICAL ASSOCIATION (2014) 311(18):1863-9. doi: 10.1001/jama.2014.3740

39. Nielsen K, Scheffer HJ, Pieters IC, van Tilborg AA, van Waesberghe JH, Oprea-Lager DE, et al. The use of PET-MRI in the follow-up after radiofrequency- and microwave ablation of colorectal liver metastases. BMC Med Imaging (2014) 14:27. Epub 20140808. doi: 10.1186/1471-2342-14-27

40. Cuda TJ, Riddell AD, Liu C, Whitehall VL, Borowsky J, Wyld DK, et al. PET imaging quantifying 68Ga-PSMA-11 uptake in metastatic colorectal cancer. Journal of Nuclear Medicine (2020) 61(11):1576-9. doi: 10.2967/jnumed.119.233312

41. Albert A, Verhaeghe J, Vangestel C, Wyffels L, Kramer G, Peeters M, et al. Optimizing ([18F] FLT) 3'deoxy-3'-fluorothymidine PET for the detection and quantification of liver metastases in colorectal cancer patients: A test-retest study. Journal of Nuclear Medicine (2016) 57.
